# Congenital malformations and medical conditions associated with orofacial clefts in children in Burkina Faso

**DOI:** 10.1186/s12887-017-0833-9

**Published:** 2017-03-14

**Authors:** Kisito Nagalo, Isso Ouédraogo, Jean-Martin Laberge, Louise Caouette-Laberge, Jean Turgeon

**Affiliations:** 1 Service of Paediatrics, El Fateh-Suka Clinic, Ouagadougou, Burkina Faso; 2 UFR/SDS, University of Ouagadougou, Ouagadougou, Burkina Faso; 3 Service of Paediatric Surgery, Charles De Gaulle Pediatric University Teaching Hospital, Ouagadougou, Burkina Faso; 4 ”Mission Sourires d’Afrique”, Montréal, Canada; 5 Department of Paediatric Surgery, The Montreal Children’s Hospital, McGill University, Montreal, Canada; 6 Department of Surgery, St Justine University Teaching Hospital, University of Montreal, Montreal, Canada; 7 Department of Paediatrics, St Justine University Teaching Hospital, University of Montreal, Montreal, Canada

**Keywords:** Cleft lip, Cleft palate, Congenital malformations, Paediatric surgery, Humanitarian surgery

## Abstract

**Background:**

Orofacial clefts are usually isolated cases but can be associated with other congenital malformations that are either recognised or unrecognised syndromes. The reported prevalence and pattern of such associated malformations, however, vary among studies. Objectives: To assess the frequencies and aetiologies of congenital malformations and associated medical conditions in children with orofacial clefts in Burkina Faso (Western Africa).

**Methods:**

A retrospective descriptive study was carried out at the El Fateh-Suka Clinic in Ouagadougou, Burkina Faso. All children who attended surgery for the repair of a cleft lip and/or palate were included in this study.

**Results:**

The frequency of congenital malformations associated with cleft lip and/or palate was 39/185 (21.1%). In the group with multiple congenital malformations of unknown origin (34 patients; 18.4%), 66.7% had cleft lip and palate, followed by isolated cleft lip (27.4%) and isolated cleft palate (5.9%). The digestive system (35.3%), the musculoskeletal system (19.6%), and eye, ear, face, and neck (15.7%) were the most affected systems. In the group of syndromic malformations (five patients; 2.7%), amniotic band syndrome (one patient), Van der Woode syndrome (one patient), Goltz syndrome (one patient), and holoprosencephaly (two patients) were identified. Medical conditions included anaemia (39.4%), infections (9.2%), malnutrition (7.5%), and haemoglobinopathies (4.3%).

**Conclusions:**

Congenital malformations and medical co-morbidities were frequent in children with OFCs. Further studies and a National Malformations Registry are needed to improve the comprehension of OFCs in Burkina Faso.

## Background

Clefts, lip and/or palate (CL/Ps) are the most common craniofacial birth defects and represent about 15% of all birth defects [1]. They occur isolated in most cases, but 15–48% of the cases are associated with other congenital malformations to constitute a syndrome or not [2–5]. The reported prevalence of congenital malformations associated with orofacial clefts (OFCs) ranges from 4.3 to 63.4% [6], the perinatal mortality rate is 228.3/1000 births [7], and the neonatal mortality rate can reach 47% of newborns with associated clefts [8]. This high burden justify the investigation of associated malformations for a better understanding of OFCs, but the results of these investigations should lead to the implementation of specific programmes for the treatment and prevention of such deformities to counter child morbidity and mortality. The medical conditions associated with OFCs are not negligible and therefore must be identified and managed to avoid jeopardising cleft repair because of anaesthetic and/or surgical complications [9, 10]. Congenital malformations associated with OFCs have been well described around the world [2, 8, 11–15]; however, in developing African countries, available data are scarce. In Burkina Faso, particularly, to our knowledge, no data is available on the morbidities associated with OFCs. The purpose of this study was thus to investigate the congenital malformations and medical conditions associated with OFCs in children in this country.

## Methods

### Study design

This study was a retrospective descriptive study of all patients seen and/or operated on under “Mission Sourires d’Afrique”, a Canadian-based Non Governmental Organisation (www.missionsouriresdafrique.com), project. Surgeries took place in 2007, 2010, and 2014 at the El Fateh-Suka Clinic in Ouagadougou, the capital of Burkina Faso (Western Africa). All patients were evaluated by a paediatrician in addition to the surgical team. All children aged between 0 and 14 years who attended surgery for CL/P repair were included in the study. Patients older than 14 years and those who had no CL/P were excluded from the study.

### Data management

The clinical and anaesthetic records, as well as patient computer database served as data sources in this study. A standardised anonymised data collection form allowed to record sociodemographic, clinical, therapeutic, complementary investigations, and outcome variables; a special attention was given to diagnoses associated with clefts. The data entry and analysis were performed by using Epi-Info7^TM^ software package (Centres for Disease Control and Prevention, Atlanta, GA, USA).

Malformations were subdivided into two groups: “isolated”, when only CL/P was present, and “associated”, when one or more additional non-CL/P malformations were recognised along with CL/P. The associated malformations were further subdivided into two categories: *recognised syndromes* and *multiple congenital anomalies (MCAs)* of unknown origin. We classified malformed systems/organs following the classification of the World Health Organization (WHO) [16]. A case could only be classified in one category.

Details of the epidemiological, clinical, and therapeutic aspects of this study have been published previously [17].

## Results

### General data

A total of 185 children consulted for CL/Ps. The clefts were associated with other congenital malformations in 39 patients, demonstrating a frequency of associated congenital malformations of 21.1%. There were 100 boys, 21 with associated congenital malformations (21.0%) and 85 girls, 18 with associated congenital malformations (21.2%).

We identified 56 congenital malformations in 39 patients (average: 1.4 malformations/patient), consisting of 51 MCAs (91.1%) and five syndromes (8.9%).

### Distribution of MCAs

Of the 185 children with CL/Ps, 34 (18.4%) had MCAs. Among the 39 patients who had associated congenital malformations, 87.2% had MCAs. Table 1 displays patterns of MCAs associated with CL/Ps in this study.

**Table 1:** Distribution of associated multiple congenital anomalies among children with orofacial clefts in Ouagadougou, Burkina Faso, in 2007, 2010, and 2014

Congenital malformation (ICD-10 code)	Type of cleft			Frequency among MCAs^d^ *n* (%)	Frequency among total sample^e^ (%)
	CL^a^	CLP^b^	CP^c^		
Digestive system (K00-K93)	6	11	1	18 (35.3)	9.7
Umbilical hernia^f^ and inguinal hernia					
Musculoskeletal system (Q65-Q79)	2	7	1	10 (19.6)	5.4
Polydactyly, syndactyly, club foot, craniosynostosis, forehead bump, and unspecified					
Eye, ear, face, and neck (Q10-Q18)	3	5	0	8 (15.7)	4.3
Cataract, strabismus, nystagmus, microphtalmy, eyelid ring, proboscis, and Tessier cleft					
Circulatory system (Q20-Q28)	2	3	0	5 (9.8)	2.7
Congenital malformations of heart					
Genital organs (Q50-Q56)	1	4	0	5 (9.8)	2.7
Cryptorchidism, hydrocele, hypospadias, and hypertrophy of the clitoris					
Nervous system (Q00-Q07)	0	4	1	5 (9.8)	2.7
Microcephaly, absence of the corpus callosum, absence of inter hemispheric fissure, epilepsy, and cerebral palsy					
Total	14 (27.4)	34 (66.7)	3 (5.9)	51 (100.0)	27.6

^a^isolated cleft lip

^b^cleft lip and palate

^c^isolated cleft palate

^d^multiple congenital anomalies

^e^ *n* = 185

^f^15 cases

### Syndromes associated with clefts

The frequency of syndromic malformations was 5/185 (2.7%). The rate of syndromic malformations among the patients with associated congenital malformations was 5/39 (12.8%). Recognised syndromes included holoprosencephaly (two cases), amniotic bands syndrome, Goltz syndrome, and Van der Woode syndrome (one case each). All five of these syndromes were observed in CLP; no syndromic cases were observed in isolated CL or isolated CP.

### Medical conditions associated with clefts

Table 2 shows the frequencies of co-existing medical conditions in children attending surgical repair for CL/Ps.

**Table 2:** Frequencies of associated medical conditions in 185 children with cleft lip and/or palate in Ouagadougou, Burkina Faso, in 2007, 2010, and 2014

Associated medical condition	Frequency	Percentage (%)
Anaemia	73	39.4
Infections	17	9.2
Malnutrition	14	7.5
Haemoglobinopathies	8	4.3

The average haemoglobin level in anaemic children was 107 ± 85 g/L (range: 55–99 g/L). An analysis of red-cell parameters identified microcytic hypochromic anaemia. Infections included upper and lower respiratory tract infections (eight cases; 4.3%), otitis and purulent conjunctivitis (three cases each; 1.6%), and skin infections, malaria, and neonatal sepsis (one case each; 0.5%). Haemoglobinopathies included six cases of sickle cell disease trait (AS), a case of homozygous sickle cell (SS), and one case of homozygous haemoglobin CC.

## Discussion

### The frequency of congenital malformations associated with CL/Ps

The frequency of congenital malformations associated with CL/Ps was 21.1%, supporting the variability of co-morbidity reported from different countries. Indeed, we found a higher frequency of associated congenital malformations, whereas African studies found lower frequencies between 4 and10.5% [18, 19]. Our result, however, was lower than in some reports from the Middle East and Asia where [20] found a frequency of 43.3% in Jordan, [2] reported 14.8% in India; [3, 4] reported 17.8 and 21.6% in Iran, respectively. Furthermore, our rates of associated malformations were even lower than those reported from Europe (29–59.2%) [13, 15, 21] and Latin America (31.4–48.4%) [5, 8]. The distribution of clefts worldwide are thus associated with geography, race, and ethnicity [22–25], suggesting that the aforementioned factors may also influence the distribution of congenital malformations associated with clefts, which may explain the differences between studies. Other sources of variability in the distribution of associated congenital malformations have also been well identified [6, 12].

We found 1.4 malformations per patient, comparable to that reported by [2] (1.8) but two-fold lower than that reported by [13] (2.7).

### The distribution of associated congenital malformations by gender

The distribution of associated congenital malformations did not differ significantly between genders, whereas [2] found a slight preponderance of boys (sex ratio 1.4) in their study where malformations were more associated with cleft lip with or without cleft palate. Our result also differed from those by [11, 14], who reported a predominance of females (sex ratio of 0.8 and 0.9 male: female, respectively) with malformations, largely associated with cleft palate. The published data, however, indicate a predominance of boys with cleft lips with or without cleft palates [15, 18, 22, 24] and of girls with cleft palates [2, 4, 5].

### The distribution of associated congenital malformations by type of cleft

Associated congenital malformations were more frequent in CLP (66.7%), comparable to the frequency of 63.4% found by [2]. Our result, though, contradicts those of other authors reporting higher frequencies of associated congenital malformations among cleft palates: [11] found that 49% of the children with associated malformations had cleft palates, [14] reported that 35.3% of malformations occurred in children with cleft palates. Many more associated congenital malformations were found among cases with cleft lip and palate than among cases of isolated cleft lip or isolated cleft palate, which was not surprising due to the higher frequency of cleft lip and palate. In addition, some authors have suggested that cleft lip and palate and a more extensive cleft may be associated with a higher risk of other congenital malformations [13, 26].

### The distribution of congenital malformations associated with clefts based on aetiology

Associated congenital malformations were mostly of unknown origin, consistent with the published data [8, 21], but their frequency varies widely among studies. Congenital malformations of the digestive system were the most frequently associated malformations (35.3% of associated malformations, 9.7% of the total sample) and consisted mostly of umbilical hernia, a common malformation in black African children [27, 28]. Congenital malformations of the musculoskeletal system were the next most common malformations (19.6% of associated congenital malformations, 5.4% of the total sample). [14] also found that skeletal malformations were secondary, with a frequency of 15.9%. [11, 24] reported frequencies of 3–6 and 22.8%, respectively. Congenital malformations of the eye, ear, face, and neck were the third most common associated congenital malformations in our study (15.7% of associated congenital malformations, 4.3% of the total sample). [2, 21] reported higher frequencies of congenital malformations associated with these organs. Congenital malformations of the circulatory system accounted for less than 10% of associated congenital malformations in our study but were more common in other studies, with frequencies of 19.6–51.4% [11, 13, 20, 21, 24]. These studies, though, diagnosed the malformations using ultrasound, which is more efficient. Genital organs were less affected by congenital malformations but were affected more often than the frequency reported by [21] (3.1%). Congenital malformations of the nervous system also accounted for less than 10% of associated congenital malformations, whereas [2, 13] reported higher frequencies of 15 and 29.2%, respectively. Finally, it is difficult to achieve unanimity on an organ or a system preferentially affected by congenital malformations associated with OFCs, and the heterogeneity of methodologies used in different studies hinders the comparison of results. Moreover, racial and ethnic differences contribute to these variations in the distribution of congenital malformations. For example, African-Americans in the United States had a lower risk of cardiac, urogenital, and craniofacial malformations compared to Caucasians but a higher risk of musculoskeletal malformations, Hispanics had a lower risk of genitourinary and gastrointestinal malformations, and Asians had a higher risk of craniofacial and musculoskeletal malformations [25].

We could clearly identify only five syndromes associated with CL/Ps because our team lacked a clinical geneticist or dysmorphologist. Syndromic cases thus most likely remained undiagnosed.

### Co-morbid medical conditions

Anaemia is a common co-morbidity in developing countries: 5.7% in Nigeria [9] and 3.6–35% in India [10, 29–31]. High frequency of anaemia in our study (39.4% of the total sample) reflects the endemicity of the condition in Burkina Faso; 58% of pregnant women, 50% of breastfeeding women, and 88% of children <5 years old are anaemic [32]. Children are not only born anaemic, but they will experience situations (prolonged breastfeeding, protein-energy malnutrition, blood loss caused by intestinal parasites, and repeated infections) that will aggravate foetal anaemia. Patients with OFCs have additional risks of malnutrition and anaemia because of feeding problems. Iron deficiency remains the leading cause of anaemia in our environment, which is usually microcytic hypochromic anaemia, as in other tropical and subtropical countries [30].

Respiratory infections were the most frequent infections. OFCs expose false food routes, so respiratory infections are frequently encountered. In our context, these respiratory infections were aggravated by adverse weather conditions due to the Harmattan, which is a cold, dry, and dusty northeasterly wind that blows over the West African sub-region from the Sahara Desert into the Gulf of Guinea between the beginning of November and the middle of March. The dust it carries is sometimes so dense that it reduces visibility and affects the health of populations, especially affecting the eyes and respiratory system and causing frequent epidemics of bacterial meningitis. In the absence of adequate protection, children with OFCs are more exposed and vulnerable to these bad weather conditions, hence more cases of colds, coughs, bronchitis, and pneumonia. Frequency (4.3%) of respiratory tract infections in our study was similar to that by [9], who found upper respiratory tract infections in 3.8% of the children with CL/Ps. Our result, however, was higher than that by [10] (2.7%) but lower than those by [29] (11.1%) and [31] (26%). The differences between studies may be due to under- or overestimation of respiratory infections; the diagnosis in our study was purely clinical. These infections must be detected and effectively treated to minimise anaesthetic and surgical risks and should not compromise the opportunity of children to receive surgery.

Malnutrition was the third most common medical condition, with a frequency of 7.5%, higher than that reported in other studies in developing countries: [9] reported 2.8% in Nigeria, [10] reported 3.6%, and [29] reported 3.9% in India. As stated above, children with CL/Ps have feeding difficulties, which can lead to malnutrition. This risk is common in all children with OFCs, but endemic undernourishment in Burkina Faso [32] constitutes a factor of increased risk of malnutrition among these children.

Our study had a few cases of haemoglobinopathies, especially the major forms of sickle cell disease. Anaesthetics and operative risks are so low that preoperative screening for sickle cell disease in children, in general and among those with CL/Ps in particular, would not be of great utility [33, 34].

Our study had some limitations due to a few previously described methodological constraints [6, 12]. For example, because of the small sample size and the hospital-based nature of this study, the results must be qualified because they do not necessarily reflect the position of congenital anomalies associated with CL/Ps in the whole population of the country. A multicentre- or population-based study will better highlight both the incidence of CL/Ps that the frequency and the nature and level of co-morbidities encountered in these orofacial defects. Despite these limitations, this study is the first of its kind in Burkina Faso, so the results should be considered as preliminary; future large-scale nationwide studies should provide more precision.

## Conclusions

This first retrospective hospital-based study in Burkina Faso has shown that congenital malformations are frequently associated with OFCs in children. The underlying and co-morbid medical conditions are not negligible in our environment and therefore must be identified and managed to ensure the success of corrective surgery of OFCs. Despite some limitations, the findings of this study can provide the groundwork for further nationwide investigations on aetiologies and risk factors associated with OFCs. We also recommend the establishment of a National OFC/Malformations Registry in Burkina Faso.

## Abbreviations

CL/P: Cleft lip and/or palate
MCA: Multiple congenital anomalies of unknown origin
OFC: Orofacial cleft
WHO: The World Health Organisation
